# Climate drivers of hospitalizations for mycoses in Brazil

**DOI:** 10.1038/s41598-019-43353-w

**Published:** 2019-05-06

**Authors:** Fabrício Brito Silva, Jessflan Rafael Nascimento Santos, Letícia Chagas da Silva, Wolia Costa Gomes, Paulo Cesar Mendes Villis, Eliane dos Santos Gomes, Edilene de Araújo Diniz Pinheiro, Conceição de Maria Pedrozo e Silva de Azevedo, Rosane da Silva Dias, Cristina de Andrade Monteiro, Julliana Ribeiro Alves Santos

**Affiliations:** 1 Mestrado em Meio Ambiente – Universidade CEUMA (UNICEUMA), São Luís, Maranhão Brazil; 2 Discente do Curso de Engenharia Ambiental – Universidade CEUMA (UNICEUMA), São Luís, Maranhão Brazil; 3 Discente do Curso de Biomedicina – Universidade CEUMA (UNICEUMA), São Luís, Maranhão Brazil; 4 Mestrado em Ciências da Saúde – Universidade Federal do Maranhão (UFMA), São Luís, Maranhão Brazil; 5 Mestrado em Gestão de Programas e Serviços de Saúde – Universidade CEUMA (UNICEUMA), São Luís, Maranhão Brazil; 6 Laboratório de Microbiologia Aplicada – Universidade CEUMA (UNICEUMA), São Luís, Maranhão Brazil

**Keywords:** Fungi, Fungal pathogenesis, Environmental impact

## Abstract

Climate can modulate human health at large spatial scales, but the influence of global, regional, and local environments remains poorly understood, especially for neglected diseases, such as mycoses. In this work, we present the correlation between climatic variables and hospitalizations for mycoses in Brazilian state capitals, evaluating the period of 2008 to 2016 at different time scales. The results indicate that climate modulates the hospitalizations for mycoses differently at annual and monthly time scales, with minimum temperature as a key climatic variable during periods of high prevalence in the 10 Brazilian capitals with the highest hospitalizations for mycoses rates. The greatest number of hospitalizations coincided with La Niña events, while a reduction was observed during El Niño events, thereby demonstrating the influence of the Pacific Interdecadal Climate Oscillation on the prevalence of mycoses in Brazil. At a regional scale, the mycoses burden in Brazil appears to respond differently to local and global climatic drivers.

## Introduction

Mycoses are neglected, under-diagnosed, and sub-estimated diseases, that represent an important public health problem, yet they are not compulsorily notifiable diseases in Brazil. Since pathogenic fungi causing systemic mycoses are found in the environment, we hypothesised that outbreaks would be affected by geoclimatic factors such as wind, precipitation, air temperature, and absolute and relative air humidity. In addition to climatic variables, the proliferation of urban pigeons and bats, drought, agricultural activity, and the practice of hunting armadillos represent sources that contribute to the risk of fungal infections and contribute to the persistence of pathogenic fungi in the environment^1–3^.

The seasonal timing of outbreaks of fungal disease can be linked to periods of greater dispersion by wind anomalies and the effect of climate change on precipitation and temperature^4^. Precipitation and temperature are crucial climatic variables for understanding the dynamics of the physical environment^5^. Understanding the seasonal behavior of these variables is fundamental for understanding the impact fungal pathogens on human health. However, there are few studies focusing on correlation between environmental variables and hospitalization due to invasive fungal infections. In addition, most of them describe only the global impact of climate change, but lack an evaluation of temporal patterns at different scales^1–3,6,7^.

In this context, epidemiology is considered an importance science which encourages research about public health issues. The influence of climate on infectious diseases as Ebola, Dengue, Chikungunya, and the West Nile fever is already well known^8^. In contrast, there is very little awareness of this phenomenon regarding mycoses.

The goal of this work was to evaluate the statistical correlations in time series (2008−2016) of climate and hospitalizations for mycoses (HM) in the state capitals of Brazil.

## Results

### Hospitalizations for mycoses in Brazilian capitals

**Figure 1:**
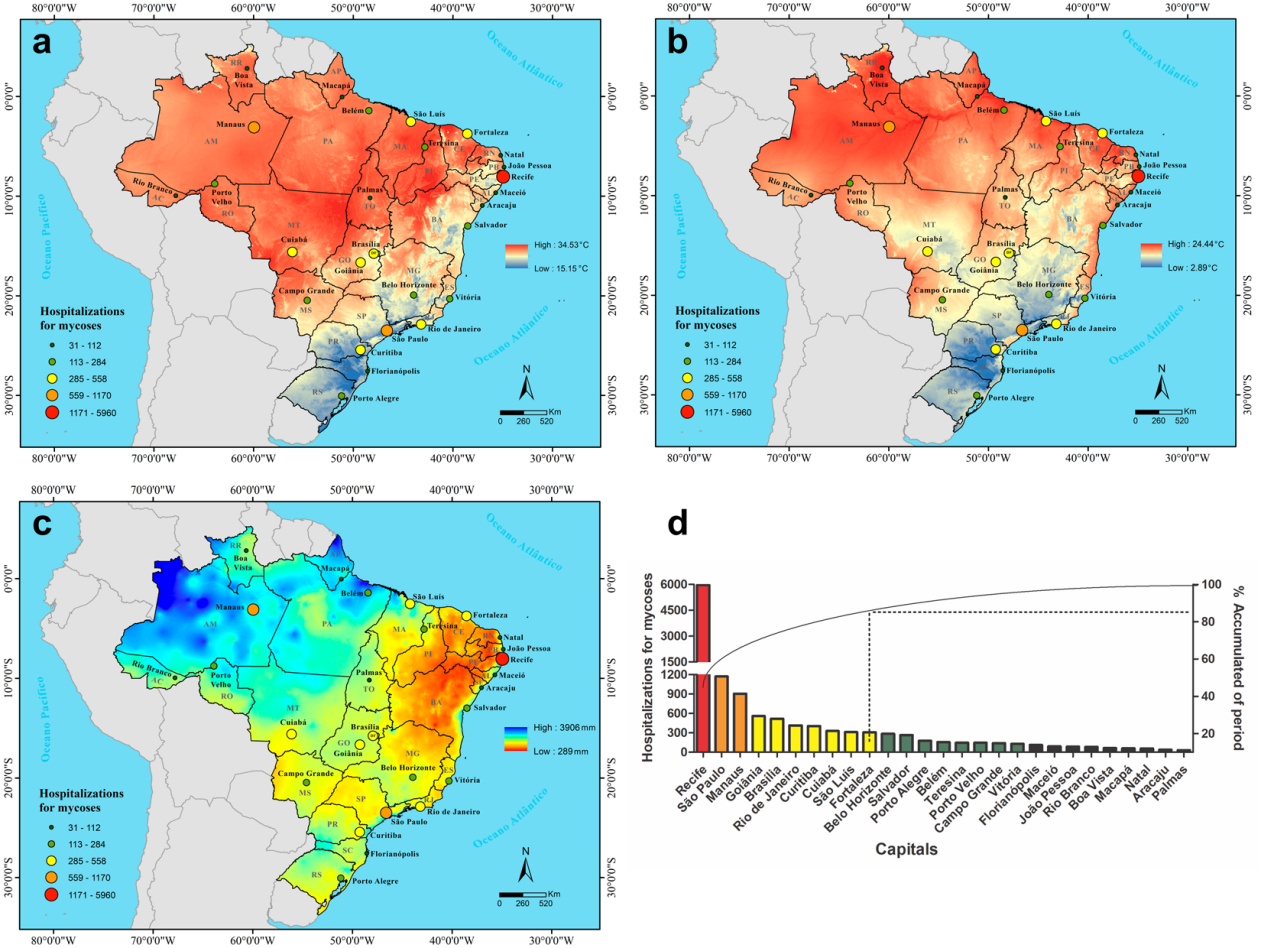
Spatial distribution of HM and maximum temperature (**a**), HM and minimum temperature (**b**), HM and precipitation (**c**), and their cumulative percentage by Brazilian state capitals between 2008 and 2016 (**d**). HM: Hospitalizations for mycoses. Brazilian States Legend: AC: Acre; AL: Alagoas; AM: Amazonas; AP: Amapá; BA: Bahia; CE: Ceará; DF: Distrito Federal; ES: Espírito Santo; GO: Goiás; MA: Maranhão; MG: Minas Gerais; MT: Mato Grosso; MS: Mato Grosso do Sul; PA: Pará; PR: Paraná; PB: Paraíba; PE: Pernambuco; PI: Piauí; RJ: Rio de Janeiro; RN: Rio Grande do Norte; RO: Rondônia; RR: Roraima; RS: Rio Grande do Sul; SC: Santa Catarina; SE: Sergipe; SP: São Paulo; TO: Tocantins. Coloured balls indicate hospitalization for mycoses.

The results show heterogeneity in the spatial distribution of HM, maximum temperature (Fig. 1a), minimum temperature (Fig. 1b), and precipitation (1c) in 26 Brazilian state capitals and Federal District and differing cumulative percentages of HM for each period (Fig. 1d).

Climatic variables differ among the different regions of Brazil. The capitals, Salvador, Aracajú, Maceió, Recife, João Pessoa, Natal, Fortaleza, Teresina and São Luís in the Northeast region; Belém, Macapá, Boa Vista, Manaus, Rio Branco, Porto Velho and Palmas in the Northern region; Cuiabá, Goiânia, Brasilia and Campo Grande in the Central-West region; and Rio de Janeiro and Vitória in the Southeast region have the highest mean values for both maximum (Fig. 1a) and minimum (Fig. 1b) temperature. On the other hand, Belo Horizonte and São Paulo in the Southeast region, and Curitiba, Florianópolis, and Porto Alegre in the South region, present the lowest mean values for maximum (Fig. 1a) and minimum (Fig. 1b) temperature. For precipitation, the highest indices were recorded in the Amazon Biome located in the Northern region of Brazil and the lowest indices found in the Caatinga biome located in the Northeast region of Brazil (Fig. 1c).

More than 80% of the reported HM occurred in just 10 Brazilian state capitals (Fig. 1d). Recife exhibited the highest proportion of cases of HM (46.08%), followed by São Paulo (9.04%), Manaus (6.99%), Goiânia (4.31%), Brasília (4.00%), Rio de Janeiro (3.20%), Belo Horizonte (2.15%), Curitiba (3.12%), Cuiabá (2.53%) São Luís (2.39%), and Fortaleza (2.38%). Interestingly, the cases in Recife were about five times higher than in São Paulo. São Paulo has 11,253,503 inhabitants, almost ten times the population of Recife with 1,537,704. However, the absolute number of hospitalizations for mycoses in Recife was about five times higher than in São Paulo, which stimulated the investigation into factors that may have favoured mycoses admissions in Brazilian capitals. In addition, both capitals have different climates and thus, different environmental conditions. In contrast, Aracaju (0.28%) and Palmas (0.23%) had markedly few cases of HM (Fig. 1d).

Overall, hospitalizations for mycoses increased annually in Brazil until 2012, and have decreased since 2013 (Supplementary Fig. 1a). However, Aracajú, João Pessoa, and Porto Velho showed an increase of HM (Supplementary Fig. 1b) from 2012 to 2014, although, these capitals have also shown a reduction in cases since 2015. Aracaju showed an increase from a maximum of 4 cases per year (2008−2013) to 17 and 11 cases in 2014 and 2015, respectively. The HM in João Pessoa increased from a maximum of 10 cases per year (2008−2012) to 23 (2013) and 17 (2014) cases. Meanwhile, Porto Velho showed an increase from a 15 cases per year (2008−2011) to 23 (2012), 26 (2013), 18 (2014), and 21 (2015) cases. These results reveal that the mycoses hospitalizations evaluated were present in all the capitals, and there are interannual HM patterns in these capitals, but not of the same magnitude.

In Aracajú, the annual average precipitation from 2008 to 2011 was 1,302.67 mm, but was 835.90 mm from 2012 to 2016. The three years of decreased precipitation in the rainy season were followed by increased HM. An increase in cases, observed in João Pessoa from January 2013 to March 2015, was also preceded by a period of reduced precipitation rates in the rainy season from 2012 to 2016. The reduction of precipitation in Aracajú and João Pessoa made them hot and less humid. In João Pessoa, the minimum temperature increased from June 2010, both in the rainy season and in the dry season (Supplementary Fig. 1b). Thus, the decrease in the temperature range during both the dry and rainy seasons coincided with the increase in HM in this period in João Pessoa.

### Multi-Time scale correlations between mycoses and climatic variables

The behaviour of the variables on the annual time scale allowed us to visualize the absolute variations in the HM in relation to the climatic variations. Since Fortaleza and São Luís showed significant correlation between mycoses and climate variables (Table 1), we compared the time series for the two capitals to evaluate the similarities between HM (Supplementary Fig. 2a), precipitation (Supplementary Fig. 2b), minimum temperature (Supplementary Fig. 2c), and maximum temperature (Supplementary Fig. 2d). Interestingly, there is a significant positive correlation observed in the behavior of HM and climate variables between the two capitals (Supplementary Table 2). These results lead us to believe that there is a similarity of these two capitals in terms of the responses of HM to climate variations.

**Table 1:** Spearman rank-order correlation of hospitalizations for mycoses with minimum and maximum temperature, and precipitation.

		Precipitation	Tmax	Tmin
Recife	Spearman R value (monthly)	0.14	−0.19	−0.24
	p value	0.12	**0.04**	**0.01**
	Spearman R value (annual)	0.28	−0.80	−0.71
	P value	0.46	**0.01**	**0.03**
Manaus	Spearman R value (monthly)	−0.01	−0.17	−0.37
	p value	0.85	0.08	**0.0002**
	Spearman R value (annual)	−0.03	−0.61	−0.86
	p value	0.94	0.08	**0.004**
Brasília	Spearman R value (monthly)	−0.004	0.25	0.20
	p value	0.96	**0.007**	**0.03**
	Spearman R value (annual)	0.26	0.40	0.38
	p value	0.49	0.29	0.31
Fortaleza	Spearman R value (monthly)	0.21	−0.26	−0.29
	p value	**0.04**	**0.01**	**0.005**
	Spearman R value (annual)	0.41	−0.60	−0.68
	p value	0.26	0.09	0.05
São Luís	Spearman R value (monthly)	0.33	−0.36	−0.34
	p value	**0.0008**	**0.0008**	**0.0007**
	Spearman R value (annual)	0.40	−0.58	−0.36
	p value	0.29	0.10	0.33

Statistically significant values (*p* < 0.05) are highlighted in bold.

**Figure 2:**
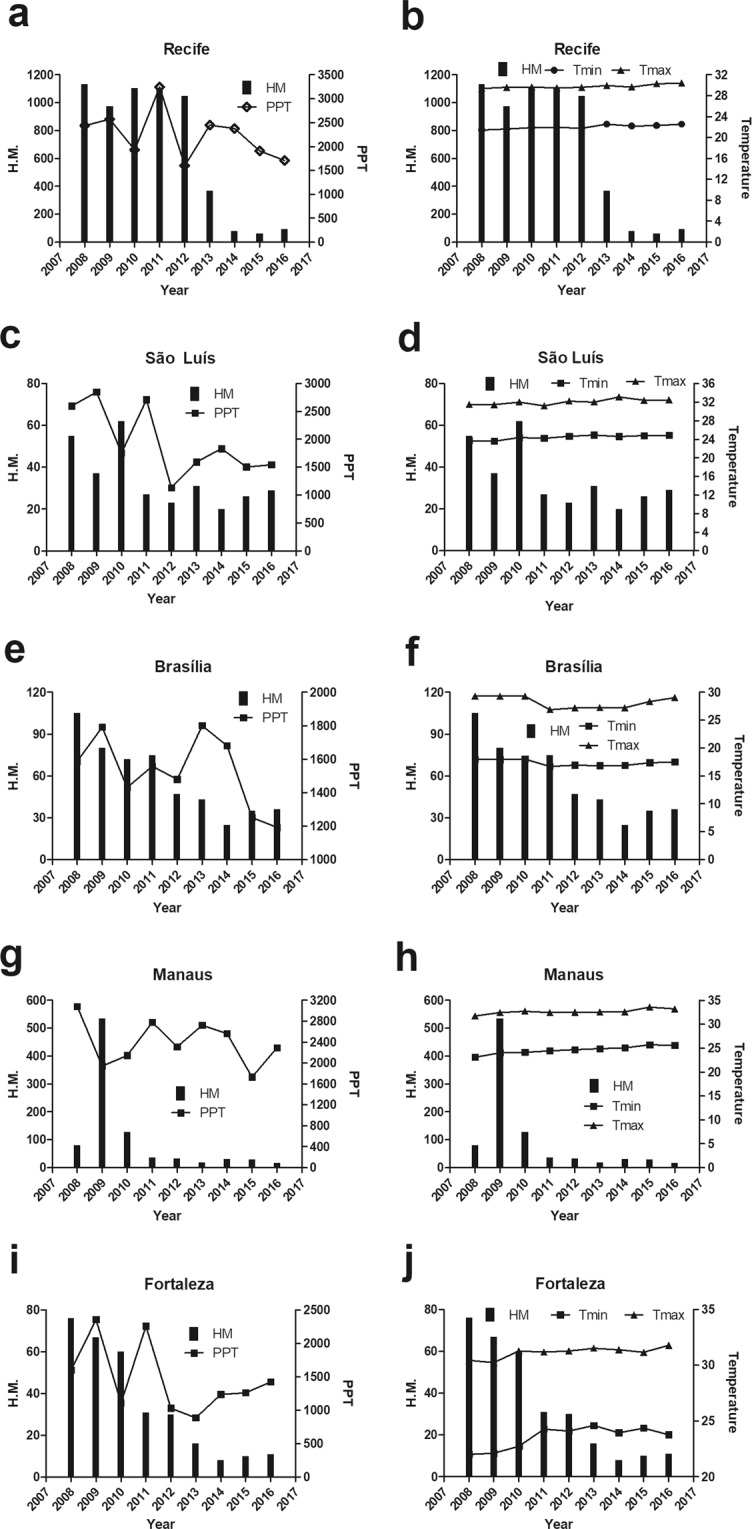
Time series of hospitalizations for mycoses and (**a**) precipitation and (**b**) minimum or maximum temperature in Recife; (**c**) precipitation or (**d**) minimum or maximum temperature in São Luís; (**e**) precipitation or (**f**) minimum or maximum temperature in Brasília; (**g**) precipitation or (**h**) minimum or maximum temperature in Manaus; (**i**) precipitation or (**j**) minimum or maximum temperature in Fortaleza. H.M.: Hospitalizations for mycoses.

The time series of HM and climate variables for states capitals that showed a significant Spearman correlation (Table 1) is presented in Fig. 2.

In Recife, there was a peak of HM in 2008 (1,133 cases) recorded at temperatures above 31 °C (Fig. 2b) and during high levels of precipitation (Fig. 2a). After 2013, there was a decrease in HM, coinciding with a reduction in precipitation levels in this state capital.

There was also a reduction in precipitation levels during the period evaluated in São Luís (Fig. 2c) and in Brasília (Fig. 2e), with a reduction in HM in periods with temperatures above 32 °C (Fig. 2d) and 30 °C (Fig. 2f), respectively.

In Manaus, during the period of high precipitation, a considerable reduction in HM was observed, followed by a peak of 534 cases in 2009, when 3,000 mm of precipitation was recorded (Fig. 2g). A low incidence of HM was observed in periods with temperatures below 24 °C and did not exceed the limit of 128 admissions registered in 2010 (Fig. 2h). A maximum of 32.6 °C was observed in 2014, when 31 cases of HM occurred (Fig. 2h).

In Fortaleza, the highest numbers of hospitalizations were recorded in 2008 and 2009 with 76 and 67 cases, respectively, reported during peak precipitation (1,602.3 and 2,359.5 mm) (Fig. 2i), with a maximum temperature above 31 °C (Fig. 2j). From 2010, fall in HM was observed, since the maximum temperature has been above 31 °C.

These results show two correlation patterns between HM and climate, one where higher HM was related to more humid and cold conditions and another where higher HM was associated with drier and hot conditions.

The Spearman rank-order correlation for the monthly and annual time scales between HM and the climatic variables are summarized in Table 1. Although not all correlation indices were statistically significant, the magnitude of the correlation on an annual scale was always higher than on a monthly scale. However, significant negative monthly correlations between minimum temperature and HM were observed in Recife (r = −0.24, p = 0.01), Manaus (r = −0.37, p = 0,0002), Fortaleza (r = −0.29, p = 0.005), and São Luís (r = −0.34, p = 0.0007). In contrast, we observed a significant positive correlation between minimum temperature and HM in Brasília (r = 0.20, p = 0.03). These results suggest a major temperature modulation of HM and provide evidence that intra-annual variations in climate can create more favourable conditions for HM, globally. Likewise, the correlation between HM and temperature was higher than between HM and precipitation.

Recife, Fortaleza, and São Luís have similar minimum temperatures, given that the average minimum temperature of these three capitals was 23.32 °C (±1.19 °C). Although the environmental conditions in Manaus differ from those in the other three capitals, the average minimum temperature recorded for all four capitals was 23.65 °C (±1.17 °C).

**Figure 3:**
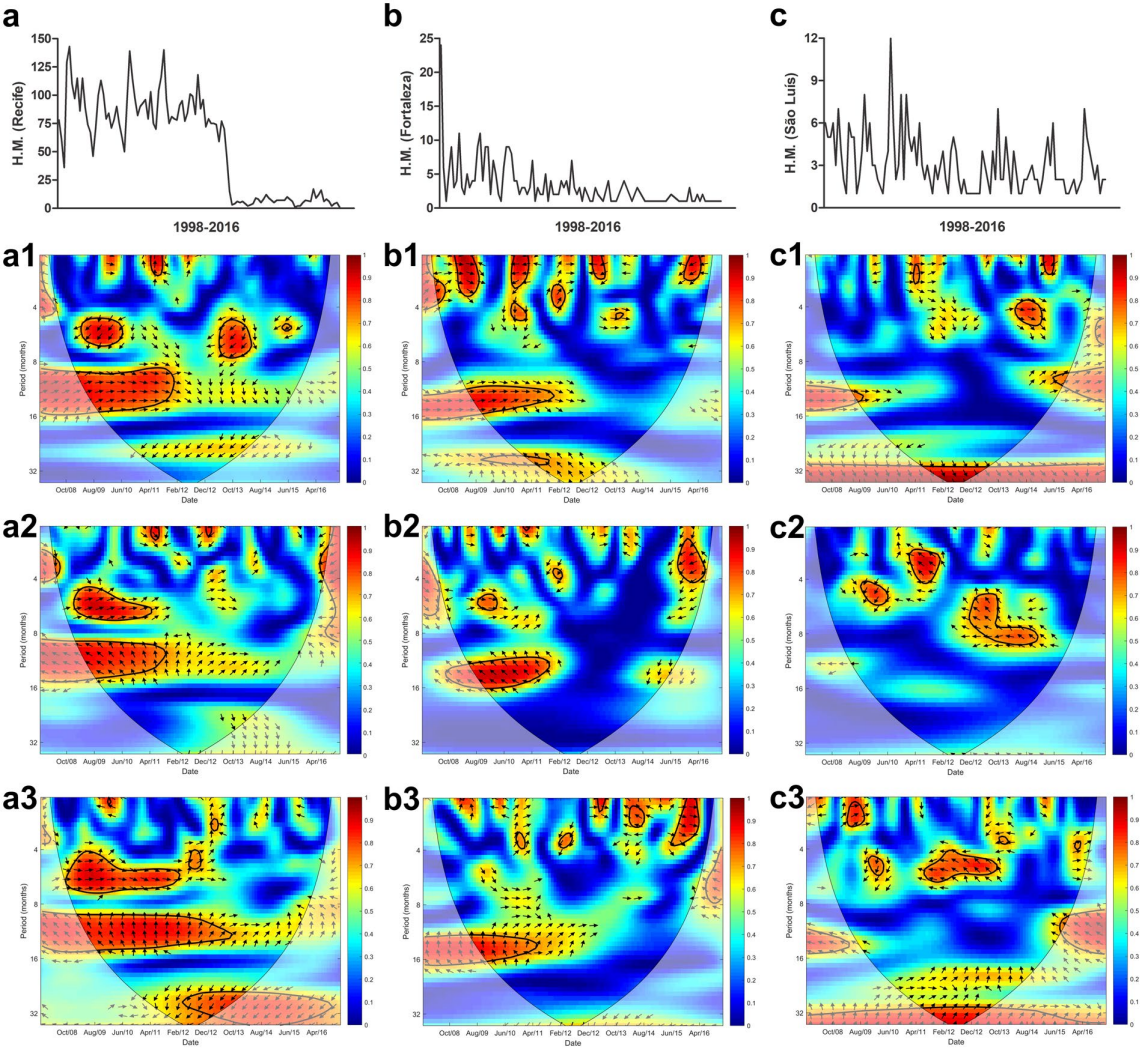
Cross wavelet coherence spectrum in (**a**) Recife for (**a1**) H.M.-precipitation, (**a2**) H.M.-maximum temperature and (**a3**) H.M.-minimum temperature, (**b**) Fortaleza for (**b1**) H.M.-precipitation, (**b2**) H.M.-maximum temperature and (**b3**) H.M.-minimum temperature and (**c**) São Luís for (**c1**) H.M.-precipitation, (**c2**) H.M.-maximum temperature and (**c3**) H.M.-minimum temperature. The colours indicate the signal power related to coherence in time-frequency space where the time series co-vary. Statistically significant regions (5% significance level) are displayed and enclosed by a solid black line and cones of influence (COI), where edge effects increase the uncertainty of the analysis, are shown as a lighter shaded region. Red regions indicate high and significant covariance within a time-frequency space. The transition from orange to blue corresponds to decreasing covariance. Arrows pointing horizontally to the right (left) indicate that the two variables are in phase (anti-), arrows pointing straight down indicate HM-leading climatic variables and arrows pointing straight up indicate climatic variable-leading HM.

**Figure 4:**
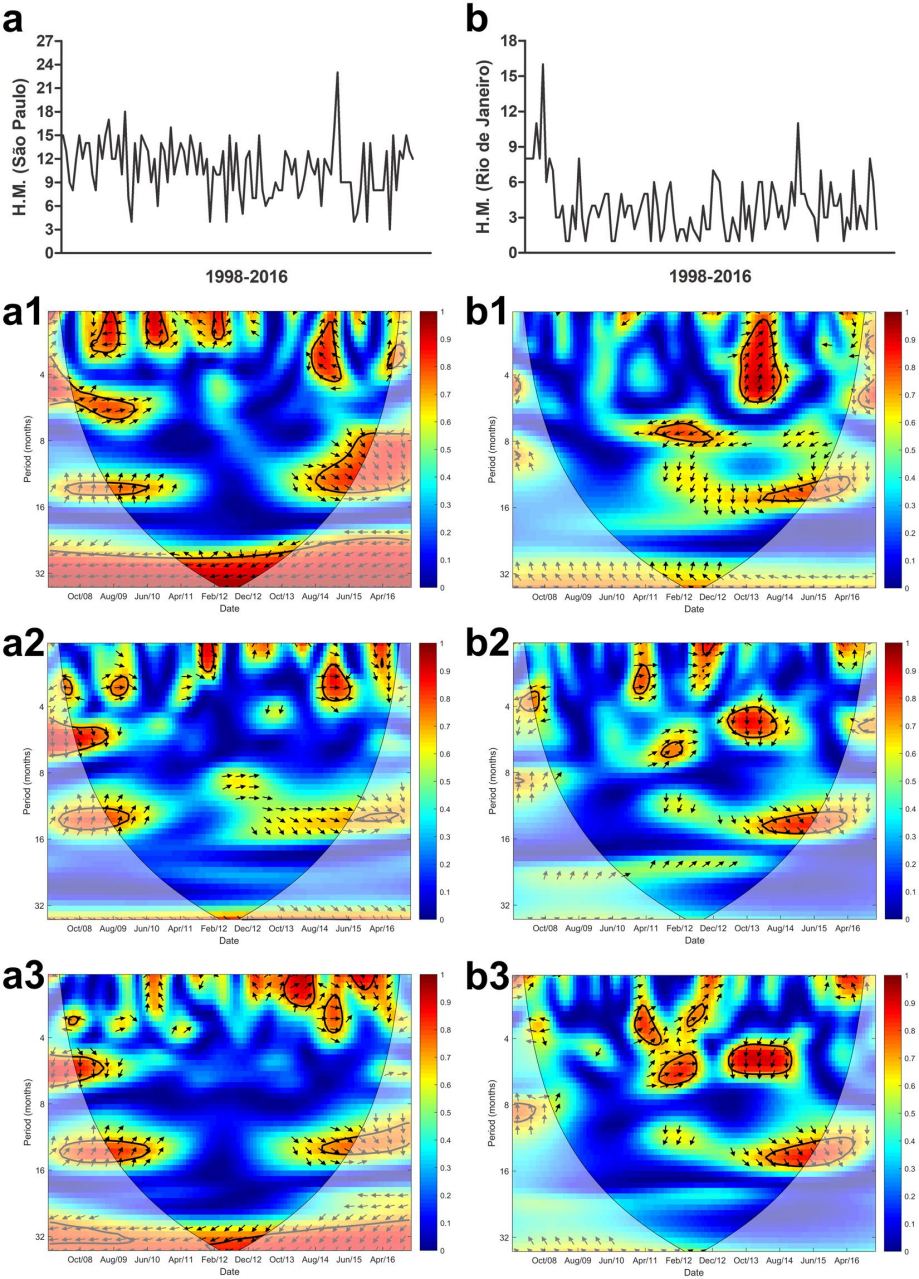
Cross wavelet coherence spectrum in (**a**) São Paulo for (**a1**) H.M.-precipitation, (**a2**) H.M.-maximum temperature and (**a3**) H.M.-minimum temperature and (**b**) Rio de Janeiro for (**b1**) H.M.-precipitation, (**b2**) H.M.-maximum temperature and (**b3**) H.M.-minimum temperature. The colours indicate the signal power related to coherence in time-frequency space where the time series co-vary. Statistically significant regions (5% significance level) are displayed and enclosed by a solid black line and cones of influence (COI), where edge effects increase the uncertainty of the analysis, are shown as a lighter shaded region. Red regions indicate high and significant covariance within a time-frequency space. The transition from orange to blue corresponds to decreasing covariance. Arrows pointing horizontally to the right (left) indicate that the two variables are in phase (anti-), arrows pointing straight down indicate HM-leading climatic variables and arrows pointing straight up indicate climatic variable-leading HM.

**Figure 5:**
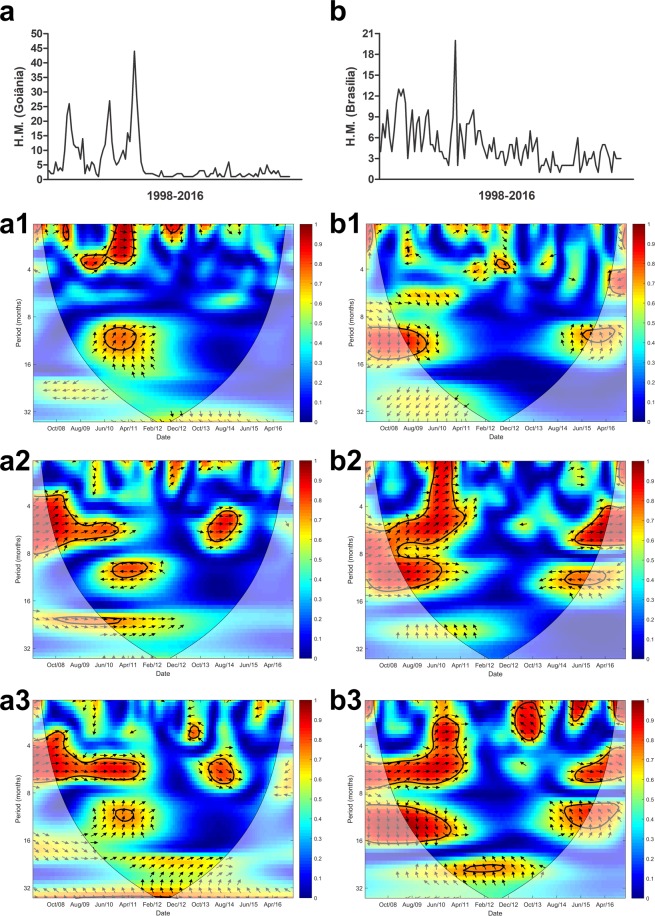
Cross wavelet coherence spectrum in (**a**) Goiânia for (**a1**) H.M.-precipitation, (**a2**) H.M.-maximum temperature and (**a3**) H.M.-minimum temperature and (**b**) Brasília for (**b1**) H.M.-precipitation, (**b2**) H.M.-maximum temperature and (**b3**) H.M.-minimum temperature. The colours indicate the signal power related to coherence in time-frequency space where the time series co-vary. Statistically significant regions (5% significance level) are displayed and enclosed by a solid black line and cones of influence (COI), where edge effects increase the uncertainty of the analysis, are shown as a lighter shaded region. Red regions indicate high and significant covariance within a time-frequency space. The transition from orange to blue corresponds to decreasing covariance. Arrows pointing horizontally to the right (left) indicate that the two variables are in phase (anti-), arrows pointing straight down indicate HM-leading climatic variables and arrows pointing straight up indicate climatic variable-leading HM.

**Figure 6:**
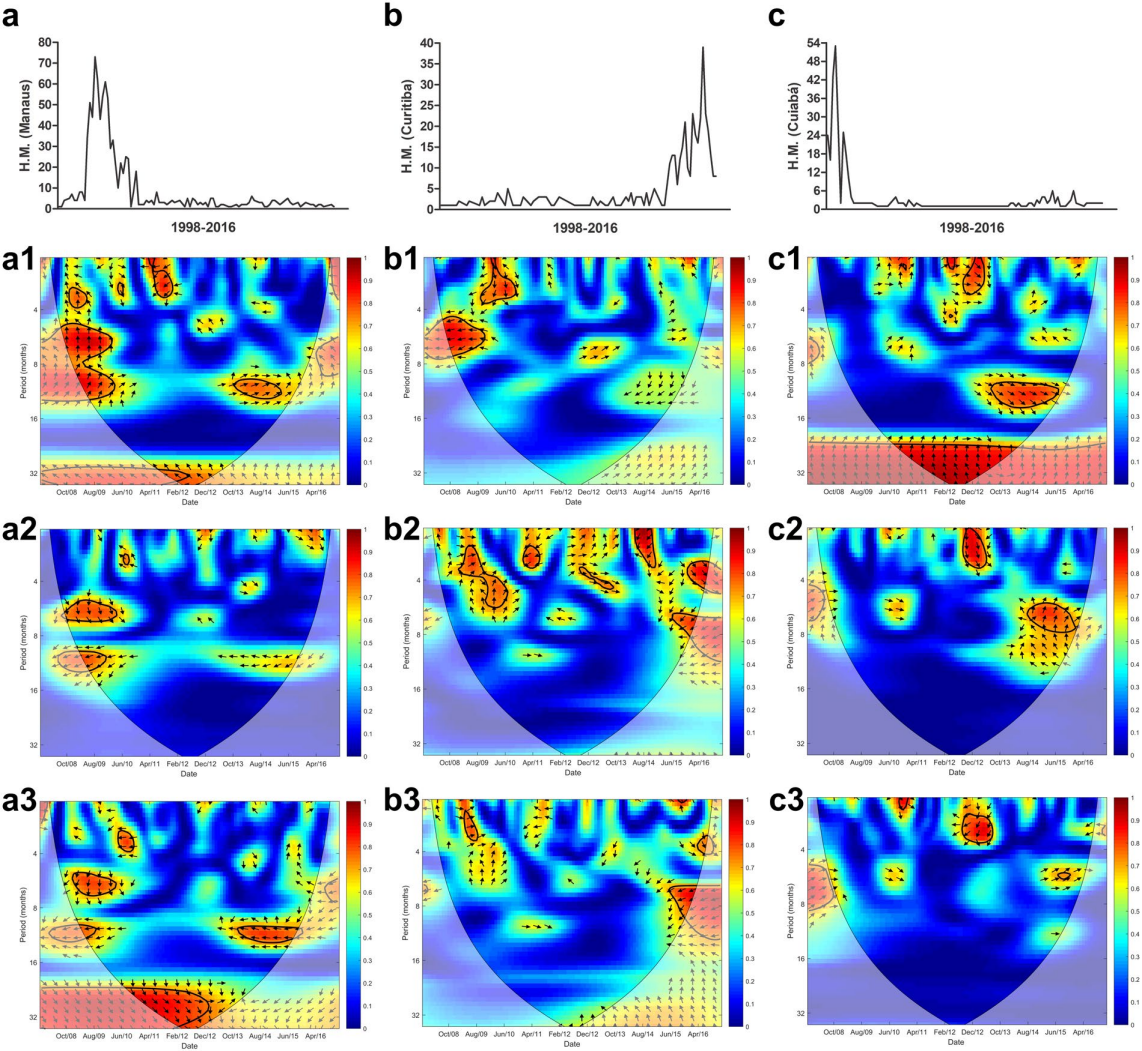
Cross wavelet coherence spectrum in (**a**) Manaus for (**a1**) H.M.-precipitation, (**a2**) H.M.-maximum temperature and (**a3**) H.M.-minimum temperature, (**b**) Curitiba for (**b1**) H.M.-precipitation, (**b2**) H.M.-maximum temperature and (**b3**) H.M.-minimum temperature and (**c**) Cuiabá for (**c1**) H.M.-precipitation, (**c2**) H.M.-maximum temperature and (**c3**) H.M.-minimum temperature. The colours indicate the signal power related to coherence in time-frequency space where the time series co-vary. Statistically significant regions (5% significance level) are displayed and enclosed by a solid black line and cones of influence (COI), where edge effects increase the uncertainty of the analysis, are shown as a lighter shaded region. Red regions indicate high and significant covariance within a time-frequency space. The transition from orange to blue corresponds to decreasing covariance. Arrows pointing horizontally to the right (left) indicate that the two variables are in phase (anti-), arrows pointing straight down indicate HM-leading climatic variables and arrows pointing straight up indicate climatic variable-leading HM.

Cross Wavelet analyses were performed in order to verify local patterns of covariance over time between HM and climatic variables in the 10 Brazilian capitals with the highest HM rates (Figs 3–6). Recife, Fortaleza and São Luís are located in the northeast of Brazil and presented similarities in HM response to climatic variables. In Recife (Fig. 3a) and Fortaleza (Fig. 3b), the annual covariance of precipitation was in-phase with HM when the prevalence of mycoses was highest. There was high covariance between HM, and minimum and maximum temperature in an annual cycle, only when rates of HM were highest, with temperature leading. In Recife, when prevalence of HM was lower, the covariance with minimum temperature was strong in a three-year cycle. In São Luís, there was a strong covariance between HM, precipitation (leading three-year cycle), and minimum temperature (Fig. 3c).

São Paulo and Rio de Janeiro are capitals located in neighboring states in the southeast of Brazil. There is a higher prevalence of HM in São Paulo (Fig. 4a) than in Rio de Janeiro (Fig. 4b), yet HM demonstrated a strong covariance with precipitation in three-year cycle throughout the studied period for both cities.

Goiânia (Fig. 5a) and Brasília (Fig. 5b) are state capitals located in neighboring states in the mid-west of Brazil, with a savanna ecosystem. In both state capitals, HM demonstrated a strong covariance in phase with maximum and minimum temperature over a semiannual cycle. Brasilia also demonstrated strong covariance between HM and minimum temperature on a semi-annual cycle (Fig. 5b).

In the group of the ten state capitals with the highest prevalence of HM, Manaus (Fig. 6a), Curitiba (Fig. 6b), and Cuiabá (Fig. 6c) presented the lowest rates. In Manaus, in the Amazon forest, when HM was at its highest, minimum temperature led covariance in a biannual cycle and precipitation was in-phase in both semiannual and annual cycles. In Cuiabá, which is in an area of savanna, the HM presented a strong covariance with minimum temperature throughout the study period.

### Pacific interdecadal climate oscillation (El Niño and La Niña) influences

**Figure 7:**
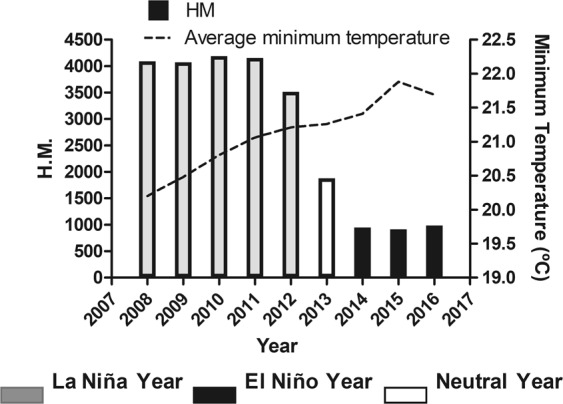
Cross-analyses of annual mean temperature from all state capitals and El Niño and La Niña occurrence in Brazil from 2008 to 2016. H.M.: Hospitalizations for mycoses.

The minimum temperature is related to nighttime temperature and is influenced by intra-annual seasonal climatic factors. In north and northeast regions, there are only two seasons: dry and wet. In contrast, from the center to south, there are the typical four seasons. In Brazil, the El Niño and La Niña are global climatic phenomena that drive the climatic variables. El Niño generally promotes drought periods and La Niña promotes periods of higher rainfall and humidity. For this reason, we cross-analyzed the annual mean temperature from all state capitals and the occurrence of El Niño and La Niña (Fig. 7). During the period of highest prevalence of HM (2008 to 2013), La Niña occurred in four out of six years. In contrast, the prevalence of HM decreased in neutral and El Niño years. The strong correlation between average minimum temperature was demonstrated by Spearman rank-order correlation (r = −0.85; p-value < 0.005).

## Discussion

We performed a retrospective detection test to verify the absolute number of HM occurrences in Brazil in time and/or space under different environmental conditions. To our knowledge, this is the first investigation about correlation between HM in Brazilian state capitals and climate variables.

Secondary databases such as *Sistema de Internações Hospitalares do Sistema Único de Saúde* (SIH-DATASUS), used in various studies in the field of collective health, are frequently used in performance analysis. Although the available information is limited to the risk adjustment of indicators, SIH is the only source with national coverage and can be of great value in the management process^9^.

The endemic mycoses in Brazil responsible for hospitalizations are coccidioidomycosis, histoplasmosis, paracoccidioidomycosis, and cryptococcosis^10,11^. Systemic mycoses are emerging infectious diseases that are neglected, and not notifiable in Brazil, with low biomedical funding allocated, despite causing high morbidity and mortality worldwide^12,13^. The mycoses data used in this study encompassed mycoses with different etiologies; beside this, the results showed a consistent temporal pattern and significant correlation with climatic variables. Reviákina *et al*.^14^ found similarities in behavior of prevalence rates of these systemic mycoses in Venezuela.

The mycoses presented periods of high and low prevalence, with similarities and differences among the Brazilian capitals. Therefore, climatic drivers on HM presented different behavior at annual and monthly scales at high and low rates of prevalence, with minimum temperature as a key climatic variable during periods of high prevalence. In this case, night temperature seems to promote different responses in HM in the savanna and Amazonian ecosystems, with the hottest night temperatures in the savanna promoting favorable conditions for fungi, rather than in wet environments, such as Amazonia. The reason may be the increase of air humidity in response to effect of the increase of temperature on soil water storage capacity^7^. This aspect could represent an important contribution to the understanding of environmental influences on the prevalence of fungal pathogens.

The status of cryptococcosis in Latin America in relation to ecology, population genetics, pathogen-host interactions, and clinical epidemiology of this mycosis was described recently by Firacative *et al*.^15^. Interestingly, Brazil leads the clinical or environmental isolation of *Cryptococcus* spp., with 45.80% of total cases. Also, the 49.50% of the environmental isolates *Cryptococcus gattii* or *C. neoformans* in Latin America were recovered from Brazil.

A study of biophysical factors carried out in Vancouver showed that the environmental concentrations of *C. gattii* in air, soil, and trees were systematically influenced by climate. Furthermore, concentrations of this fungus in the environment peaked in periods with moderate intensity of winds, leading to an increased risk of exposure during the summer. The high temperatures were associated with low fungal burden in environment^3^.

The lag time should be evaluated because there is a latency period related to the infection and disease onset that can range from months to years depending on the mycoses. In addition, the diagnosis may be delayed, since the symptoms are often non-specific and invasive mycoses may be confused with other infectious diseases^10^. Barrozo *et al*.^7^ observed a close relationship between climatic factors and the incidence of the acute/subacute form of paracoccidioidomycosis in Botucatu (São Paulo), an endemic area for this disease. The incubation time can last for up to 2 years before the probable exposure time, depending upon the levels of soil water storage, showing the relationship between *P. brasiliensis* and greater absolute precipitation/humidity^6^. Calle *et al*.^16^ also suggested a possible correlation between the cases of paracoccidioidomycosis and annual precipitation in the year prior to diagnosis, for cases in Colombia between 1970 and 1999.

In the South America, the temperature and precipitation at a local scale are influenced by the Pacific Interdecadal Climate Oscillation (El Niño and La Niña). In northern South America, i.e. most of Brazil, and countries such as Venezuela, El Niño promotes dry climatic conditions in contrast to La Niña that is associated with wet climatic conditions. However, in other countries, such as Argentina and Ecuador, this connection is reversed^17^. Thus, global events such as El Niño and La Niña have different influences on local climate in South America.

The relationship between climate, prevalence of mycoses, and the Pacific Interdecadal Climate Oscillation were raised in other studies, but without evaluating the statistical correlations in time series at different time scales^7,18,19^. In our study, the period of nine years (2008–2016) was sufficient to comprise five years of La Niña, one year of neutrality and three years of El Niño. The greatest number of cases of HM occurred between 2008 and 2013, coinciding with La Niña events. In contrast, the reduction of HM occurred in the last 3 years (2014–2016), coinciding with El Niño events. This result makes it clear that the Pacific Interdecadal Climate Oscillation influences mycoses in Brazil.

The relationship between the Pacific Interdecadal Climate Oscillation and epidemiological events has been widely reported^20–25^. However, there are few studies involving responses of epidemiological events from mycoses to climatic variations, using long time series, as showed by Barrozo *et al*.^7^. These authors demonstrated the influence of El Niño on one temporal series of paracoccidioidomycosis, despite only studying one endemic region in the center-west region of the state for this mycosis.

Besides the influence of climatic anomalies, such as greater precipitation and humidity, activities such as expansion of agricultural land, deforestation of areas for agriculture and livestock, and migratory flows from the other states coincided with the geographical distribution of paracoccidioidomycosis in some brazilian states, as Maranhão^26^.

Although precipitation often influences the levels and distribution of fungi in the environmental, the presence of *Coccidioides* spp. in soil can be influenced by the absence of precipitation by sporulation and easy spread of fungi. Several studies have shown a negative correlation between the seasonality of cases and precipitation, as support for these phenomena. Although fungi may not be directly influenced by precipitation, it may be affected by conditions in the environment that produce favorable conditions of growth fungus^27^. There were three cases of coccidioidomycosis in Sobral (Ceará) in a hot period^28^. Shriber *et al*.^29^ showed in Arizona and in California that counties with high climate variability are more vulnerable to outbreaks of coccidioidomycosis. Recently, Araújo *et al*.^30^ reported cases of coccidioidomycosis in Pernambuco (Brazil), a region for which the disease had never been reported previously, and their possible association with the recent drought that occurred in the region. As with other systemic mycoses, temporal fluctuations in the incidence of disseminated histoplasmosis can also be explained by climatic variations^31^.

Quantifying the role of climate variability in the mechanisms of forcing and feedback between climate and mycoses remains an important unresolved problem, the study of which is necessary to understand fungal ecology. This study highlights that climate influences the different responses to HM in state capitals of Brazil in both outbreak and non-epidemic situations, mainly influenced by the minimum temperature. Also, we considered climatic anomalies represented by La Niña and El Niño to be important phenomena for a better understanding of the consequences of climatic change on the incidence of mycoses in Brazil. We highlight the importance of geospatial information in the surveillance of endemic mycoses by the development of a model for spatiotemporal prediction, based on environmental variables, for intervention and prevention of diseases caused by fungi that can be modulated by the climate. The surveillance of mycoses in Brazil can be used to inform more vulnerable regions and predict the seasonal occurrence of climate-driven mycoses, thereby allowing more efficient use of public resources.

## Methods

### Study area

Brazil is a continental country and the largest country in South America, with 209,091,326 habitants distributed across 26 federal states, and the Federal District. This country covers an area of 8,515,759.090 km^2^ and has a population density of 22.43 inhabitants/km^2^. Brasília is the capital of Brazil, but São Paulo is the most populated city with 12,176,866 inhabitants. The country has a wide diversity of climates with 5 biomes (Amazonia, Savanna, Caatinga, Pantanal, Atlantic Forest and Pampa) and 6 climatic types (Subtropical, Tropical Atlantic, Elevated tropical, Semi-arid, Tropical and Equatorial). Approximately 76.0% of the population live in urban areas.

### Mycoses and climate database

We constructed a time series of hospitalizations for mycoses and climate data in 26 Brazilian state capitals and the Federal District. All federal units in Brazil have a reference laboratory*, the Central Public Health Laboratory (LACEN) -* Ministry of Health. Secondary data regarding the absolute number of hospitalizations for approved mycoses of residents, counted as hospitalizations in the period were obtained from the database of the *Sistema de Internações Hospitalares do Sistema Único de Saúde* (SIH-DATASUS). The four most important endemic systemic mycoses in Brazil responsible for hospitalizations are coccidioidomycosis, histoplasmosis, paracoccidioidomycosis, and cryptococcosis.

Climatic data (precipitation and minimum/maximum temperature) were obtained from 27 meteorological stations presented in each capital and operated by the National Institute of Meteorology (INMET). Monthly and annual records of HM, precipitation levels, and maximum and minimum temperature were used in the period from 2008 to 2016. These data made it possible to understand the spatial-temporal evolution of changes in land use its interaction with the climate. The data collected and clustered in this stage were processed and stored in a Geographic Information System (GIS).

### Statistical analysis

We performed a Spearman rank correlation analysis with our health and environmental data using GraphPad Prism version 5.0.

We performed a Spearman rank correlation analysis, without corrections for multiple comparisons, for HM based on climatic variables (precipitation and maximum/ minimum temperature) at monthly and annual time scales for each region.

### Wavelet analysis

The Cross Wavelet Analysis (CWA) is a technique based on the cross‐spectrum and wavelet transform analyses which allows us to compare two-time series and identify synchronous periods^20,32^. The coherence spectra derived from CWA closely resemble the traditional correlation coefficient, and it is useful to think of the wavelet coherence as a localized correlation coefficient in the time frequency space^33^. An advantage of coherency spectral on the traditional correlation coefficient is that asynchronous periods compromise the traditional correlation analyze decreasing the coefficient and hiding periods of high covariance, for example in outbreak situations. Another advantage is the possibility of to analyze lag-lead or phase relationships between two time series, where a variable first initiates a variation modulating the behavior of the other variable.

In this work we performed the Morlet Wavelet Transform to compute the coherence spectra using precipitation, maximum and minimum temperature and HM monthly data, from 2008 to 2016. The data were pre-processed according to a previously described method^32,33^ to identify the significant periods for each variable, the cross wavelet power to identify periods where HM-precipitation and HM-maximum or minimum temperature showed a high common power, and the coherence spectra to identify the local co-variability of HM-precipitation and HM-maximum or minimum temperature. Statistically significant regions (5% significance level) are displayed in the Figs 3–6 and enclosed by a solid black line and the cones of influence (COI), where edge effects increase the uncertainty of the analysis, are shown by the lighter shaded region. Red regions indicate high and significant covariance within a time–frequency space. The transition from orange to blue corresponds to decreasing covariance. Arrows pointing horizontally to the right (left) indicate that the two variables are in phase (anti-), arrows pointing down indicate HM-leading climatic variables, and arrows pointing up indicate climatic variable-leading HM.

## Supplementary information

Supplementary Figure 1, 2, and 3, and Supplementary Table 2.

## Data Availability

All data generated or analysed during this study are included in this article (and its Supplementary Information files). Hospitalizations for mycoses data from the *Sistema de Internações Hospitalares do Sistema Único de Saúde* (SIH-DATASUS) were used. This data is also available to other researchers in http://www2.datasus.gov.br/DATASUS/index.php?area=0203&id=6926.
