# Supplementary material for: Climate drivers of hospitalizations for mycoses in Brazil
Source: Sci Rep. 2019 May 6;9:6902. doi: 10.1038/s41598-019-43353-w (PMC6502841; doi:10.1038/s41598-019-43353-w)
Supplement: Supplementary file 1 — Supplementary Figure 1, 2, and 3, and Supplementary Table 2. [file 41598_2019_43353_MOESM1_ESM.doc]

**Climate drivers of hospitalizations for mycoses in Brazil**

Fabrício Brito Silva^1^, Jessflan Rafael Nascimento Santos^1^, Leticia Chagas da Silva^1^, Wolia Costa Gomes^1^, Paulo Cesar Mendes Villis^1^, Eliane dos Santos Gomes^2^, Edilene de Araújo Diniz Pinheiro^3^, Conceicao de Maria Pedrozo e Silva de Azevedo^4^, Rosane da Silva Dias^5^, Cristina de Andrade Monteiro^6^, Julliana Ribeiro Alves Santos^1*^

**Supplementary Material**


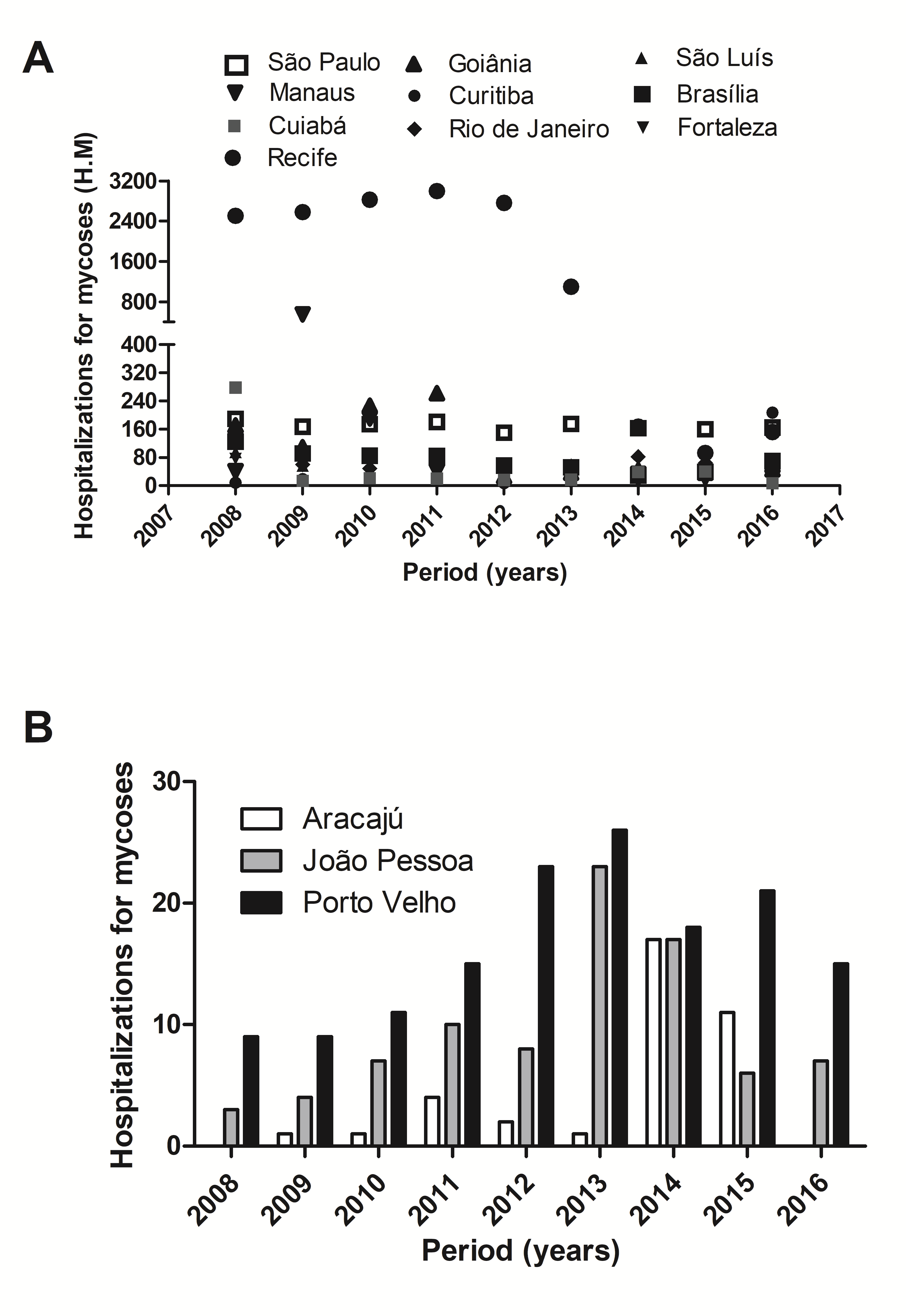


**Supplementary Figure 1.** Hospitalizations for mycoses in the first 10 Brazilian state capitals **(a)**. The three capitals which showed an increasing in hospitalization for mycoses from 2012 to 2014 **(b)**.


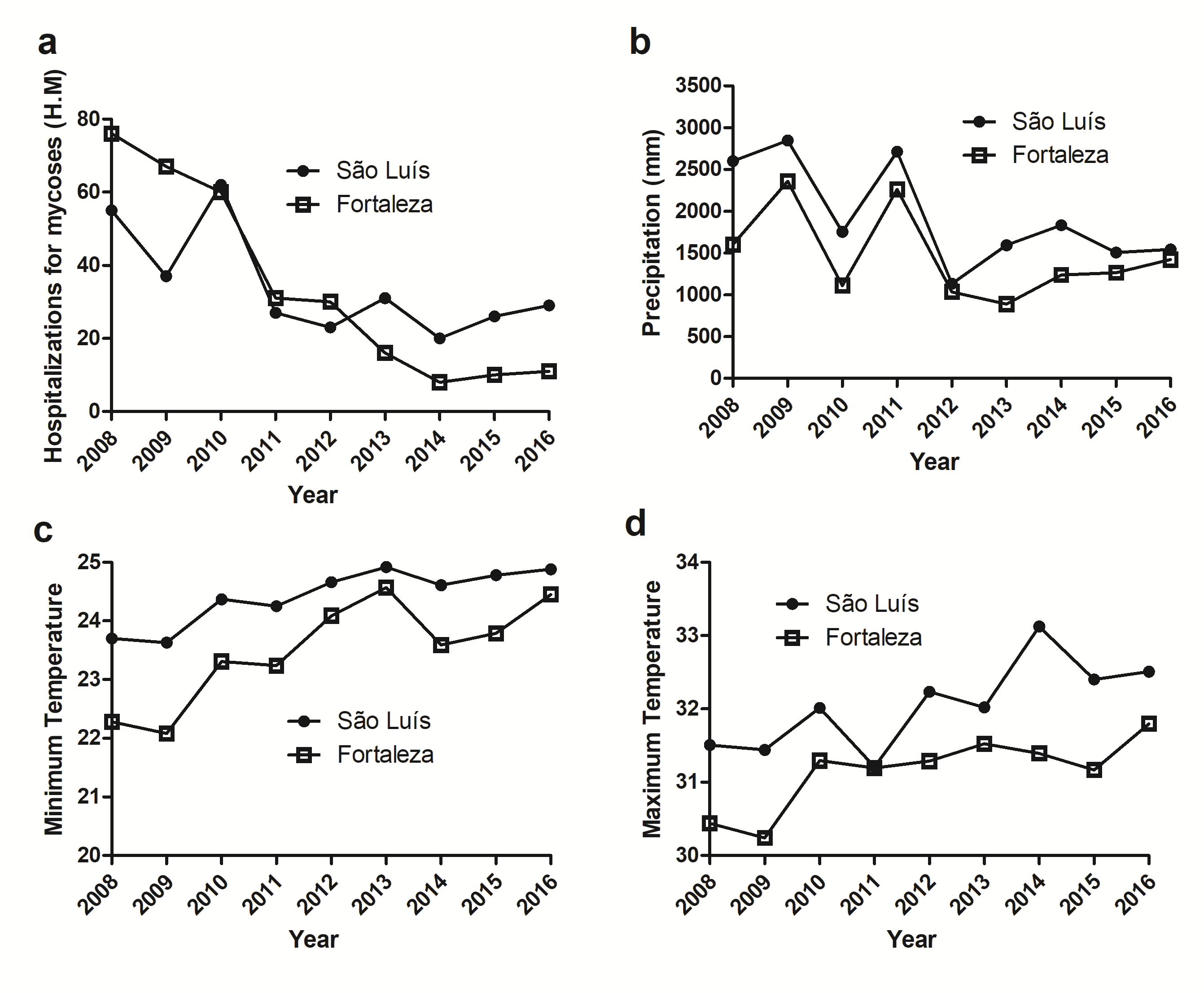


**Supplementary Figure 2.** Time series for (a) hospitalizations for mycoses; (b) precipitation; and (c) minimum and (d) maximum temperature in Fortaleza and São Luís.

**Supplementary Table 2.** Spearman rank-order correlation of São Luís and Fortaleza regarding hospitalizations for mycoses, precipitation, maximum and minimum temperature.

|  | Hospitalizations for mycoses | Precipitation | Maximum Temperature | Minimum Temperature |
| --- | --- | --- | --- | --- |
| Spearman R value | 0.35 | 0.88 | 0.69 | 0.83 |
| p value | 0.0015 | <0.0001 | <0.0001 | <0.0001 |

**
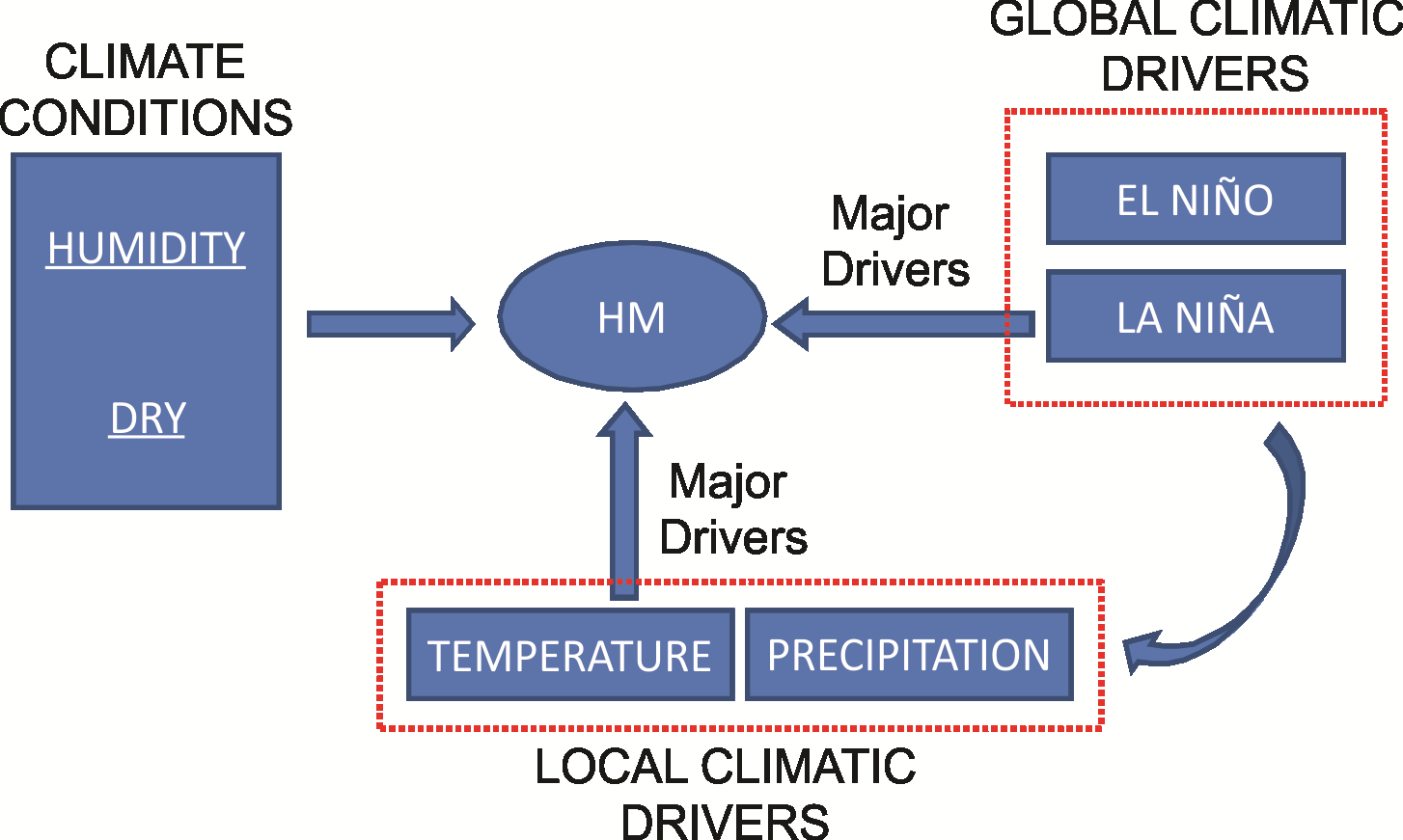
**

**Supplementary Figure 3.** Final diagram with the main results
